# Before and after: The impact of the Roe v. Wade overturn on prenatal genetic counseling practice

**DOI:** 10.1002/jgc4.70088

**Published:** 2025-08-05

**Authors:** Elizabeth J. C. Hart, R. Beth Dugan, Anne C. Heuerman, Beverly M. Yashar

**Affiliations:** ^1^ Department of Human Genetics University of Michigan/Michigan Medicine Ann Arbor Michigan USA; ^2^ Genetic Counseling Graduate Program University of Michigan Ann Arbor Michigan USA; ^3^ Molecular Genetics, Department of Maternal Fetal Medicine Indiana University Health Indianapolis Indiana USA; ^4^ Women's Health Natera Inc. San Carlos California USA

**Keywords:** abortion, Dobbs v. Jackson, genetic counselors(ing), pregnancy, prenatal diagnosis, Roe v. Wade, termination

## Abstract

Genetic counselors (GCs) play key roles in discussing pregnancy management options with patients, including abortion care and coordination. Since the 2022 overturn of *Roe v. Wade*, total or near‐total abortion bans and frequent ban switching have occurred in many US states. A mixed methods approach (27 question survey assessing prenatal GCs' abortion referral/coordination practices before and after the overturn and semi‐structured qualitative interviews exploring counseling adaptations, emotional well‐being, and job satisfaction) was used to assess the effects of the overturn on prenatal GC practice. Survey responses from 35 prenatal GCs who have practiced for over 1 year were analyzed in four state categories that considered type and number of gestational age restriction changes after the overturn: Stable Restrictive (SR; *N* = 5), Newly Restrictive (NR; *N* = 5), Unstable (US; *N* = 8), and Stable Permissive (SP; *N* = 17). Paired *t*‐tests compared “before” to “after” responses, and one‐way ANOVA and Tukey tests compared differences between state categories. Results showed a statistically significant increase in reported distance to the nearest abortion clinic (*p* = 0.002) and reported wait times for abortion appointments (*p* < 0.001). Additionally, we found a statistically significant increase in prenatal GCs reporting that they often or always refer patients out of state for abortion care (*p* = 0.009). The reflexive thematic analysis framework was used for qualitative analysis. Through deductive and inductive analysis of follow‐up interviews with 12 prenatal GCs from each state category (SR *N* = 2; NR *N* = 2; US *N* = 3; and SP *N* = 5), the researchers produced three cross‐cutting themes: GCs altered practice due to legal uncertainty and changing policies, GCs built resource networks and self‐educated, GCs experienced increased burden from their role in abortion coordination and care. These findings emphasize prenatal GCs' challenges in supporting their patients in all pregnancy management options and highlight the adaptations they have made to contend with abortion restrictions after the Roe v. Wade overturn.

**What is known about this topic**

Previous studies of genetic counselors with prenatal experiences found the majority of participants had concerns regarding the impacts of abortion restrictions on patient choice in the prenatal genetic counseling setting (Jayaraman et al., 2021). Prenatal GCs working in restrictive states perceive a negative impact of abortion restrictions on their patients due to the increased burden of seeking abortion and counsel their patients differently in the setting of abortion restrictions (Cooney et al., 2017; Jayaraman et al., 2021; Koenig et al., 2019).

**What this paper adds to the topic**

This exploratory, mixed methods study examined prenatal genetic counselors' abortion coordination/referral practices and counseling changes across multiple states after the overturning of Roe v. Wade, an unprecedented nationwide change in abortion access policy.

## 1 INTRODUCTION

According to the Guttmacher Institute, the United States has trended more hostile to abortion over the last two decades (Guttmacher Institute, 2019). 2021 was a record‐breaking year for laws restricting abortion access, with 663 restrictive provisions introduced, 108 of which were passed and enacted (Guttmacher Institute, 2019). Restrictions enacted by states, including gestational age bans that set legal limits on abortion by weeks of pregnancy, were part of decades‐long efforts by opponents of abortion to overturn the 1973 Roe v. Wade ruling, which protected a pregnant person's right to have an abortion without certain government interference (Roe v. Wade, 1973). Roe v. Wade protected a person's right to have an abortion up to the point of fetal viability, the time at which a fetus would be considered able to survive outside of the uterus. The American College of Obstetricians and Gynecologists (ACOG) considers fetal viability to be between 20 weeks 0 days and 25 weeks 6 days of gestation, with an approximately 50% survival rate for structurally normal fetuses born at 24 weeks, although advancements in premature neonatal intervention may be lowering this threshold since the time of this publication (ACOG, 2002). States have used their own interpretations of viability in their laws, with the majority settling on 22‐ to 24‐week cutoffs following the initial Roe v. Wade (1973) and Planned Parenthood of Southeastern Pa. v. Casey (1992) (Guttmacher Institute, 2020).

Of the many challenges to reproductive rights in the United States in 2021, two were distinctly impactful: (1) the state of Texas enacted a 6‐week abortion ban with new, specific language that made it significantly more difficult to challenge in court and (2) the state of Mississippi appealed a court case over the constitutionality of its 15‐week abortion ban, which was blocked at the time. Texas's 6‐week ban was allowed to remain in effect and other states began using copycat legislation; the Mississippi lawsuit became the subject of the Dobbs v. Jackson Women's Health Organization case heard by the Supreme Court at the end of 2021. In its brief, the state of Mississippi directly asked the Supreme Court to overturn Roe v. Wade. A leak of the draft case decision in May 2022 indicated Roe v. Wade would be overturned (Gerstein & Ward, 2022). After the leak, states quickly moved to enact total bans, trigger bans, and 15‐week bans in the time leading up to the final decision (Guttmacher Institute, 2022a, 2022b, 2022c). On June 24, 2022, the Supreme Court decided in favor of Dobbs, officially overturning Roe v. Wade and nearly 50 years of precedent (Dobbs v. Jackson Women's Health Organization, 2022).

The Roe v. Wade overturn is the most significant change in abortion access policy in the history of modern genetic counseling. Just 3 days after the Roe v. Wade overturn nine states had a total abortion ban and one state had a 6‐week ban (Guttmacher Institute, 2022a, 2022b, 2022c). Legal challenges were made to state‐level bans across the nation in the days following the overturn, temporarily blocking some bans. The legality of abortion has changed frequently in some states since Roe v. Wade was overturned due to lawsuits over the constitutionality of state‐level bans (McCann, 2022). These changes occur as the lawsuits proceed and rulings at various points either block the ban or reinstate it until a final decision is made. At the time of this writing in January 2025, 18 states have gestational age bans restricting the legal time frame of abortion to 15 weeks or fewer; of those 18 states, 16 have total or near‐total abortion bans (Guttmacher Institute, 2025).

Historically, it is within genetic counselors' (GCs) scope to “discuss the features, natural history, means of diagnosis, genetic and environmental factors, and management of risk for genetic/medical conditions and diseases” (NSGC, 2024). Per a 2022 position statement from NSGC regarding access to reproductive healthcare, this extends to discussion of pregnancy management options including abortion with patients in the wake of a fetal diagnosis (NSGC, 2022). Many factors contribute to the point in a pregnancy when a patient learns of the risk of a genetic diagnosis, particularly the timing of imaging and testing. Fetal anatomy ultrasounds have historically been performed between 18 and 22 weeks of gestation (ACOG, 2018). For findings such as heart defects or central nervous system abnormalities, fetal echocardiogram and MRI may be recommended (if available) for clarification of diagnosis and prognosis, but additional studies take time to coordinate (Carvalho et al., 2023; Society of Maternal Fetal Medicine, 2020). In other scenarios, patient and reproductive partner testing may be required prior to subsequent fetal diagnosis, often spanning several weeks. The timing of diagnosis juxtaposed with abortion legal limits leads to a challenging timeline for prenatal GCs supporting patients in making informed, non‐rushed decisions. In the face of abortion restrictions, counselors report discussions of abortion earlier than they had in the past and felt that they had to be more directive about timing and decision‐making in order to keep abortion options open for patients (Koenig et al., 2019).

Studies prior to the Roe v. Wade overturn have examined the effects of statewide changes in abortion access on prenatal genetic counseling. Differences in counseling were identified between states with policies hostile to abortion versus supportive of abortion, including the frequency of discussion of specific topics such as options for pregnancy management other than pregnancy termination, waiting periods, and traveling for abortion care (Jayaraman et al., 2021). A 2021 study found that prenatal GCs practicing in Ohio, a state with frequently proposed restrictions, had trouble keeping up with which legislation was in effect and what services they could legally provide under the laws (Heuerman et al., 2022). Studies since the Roe v. Wade overturn have documented its effects on reproductive healthcare provision, including increased fear due to uncertainty about the consequences of providing care under restrictions (Czarnecki et al., 2023). Some providers noted their institution implemented policies more restrictive than those required by the state laws after those state laws led the institution to examine their own practices (Czarnecki et al., 2023).

Given the magnitude and novelty of changes in the abortion access landscape as a result of the Roe v. Wade overturn, it is vital to study and document the impacts of the overturn. While previous research provides a window into the effects of abortion restrictions on prenatal genetic counseling, this study introduces themes that cut across prenatal genetic counseling practices in this new setting.

## 2 METHODOLOGY

Approval to conduct this human subjects' research study was obtained by the University of Michigan Institutional Review Board (IRB# HUM00227742). All procedures followed were in accordance with the ethical standards of the responsible committee on human experimentation (institutional and national) and with the Helsinki Declaration of 1975, as revised in 2000. Informed consent was obtained from each study participant.

### 2.1 State categorization framework

**FIGURE 1:**
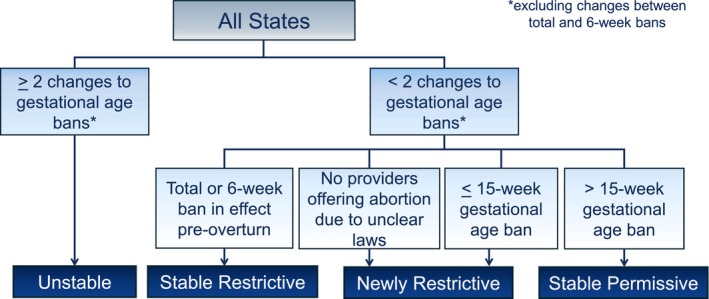
State categorization framework.

We developed a novel state categorization framework to capture the evolution of state legislation across the United States and understand cross‐cutting experiences and perspectives. The primary researcher, EH, has a background in reproductive health policy specifically pertaining to abortion access policy. Her prior understanding of state‐level abortion access policy in the United States assisted in the creation of the state categorization framework. The framework categorizes states based on the type and number of gestational age restriction changes since the overturn, which we tracked using the Guttmacher Institute's abortion law updates (Guttmacher Institute, 2023a, 2023b). There are four categories: Unstable (US), Stable Restrictive (SR), Newly Restrictive (NR), and Stable Permissive (SP) (Figure 1). Fifteen weeks was chosen as the gestational age cutoff distinguishing between restrictive versus permissive states. Restrictive states were split into two categories based on whether states had a total or near‐total ban in place prior to the overturn. We have not reported which states are included in each category to maintain anonymity and reduce legal risk for participants, particularly those practicing in states with very few prenatal GCs.

All states in the United States were categorized based on the type and number of changes to gestational age restrictions. First, states with 2 or more gestational age (GA) bans after June 24, 2022, were identified. States with 2 or more changes to GA bans were categorized as Unstable (US). Those with fewer than two changes were subcategorized into four groups based on the cutoff of the GA ban in weeks: Stable Restrictive (SR) states had total or 6‐week bans prior to June 24, 2022, Newly Restrictive (NR) states had a ≤15‐week GA ban or providers stopped offering abortion care after June 24, 2022, and Stable Permissive (SP) states had a >15‐week GA ban after June 24, 2022.

### 2.2 Eligibility and recruitment

Certified GCs who were currently practicing in a clinical prenatal setting, had over 1 year of experience in the specialty, and provided referrals for abortion care were eligible to participate in the study. Study participants were recruited through the National Society of Genetic Counselors (NSGC) Research Survey E‐Blasts and emails sent to genetic counseling program directors for distribution in target states from the Unstable and Restrictive categories. The Research Survey E‐Blast alone did not prompt enough responses from GCs in SR, NR, or US states, so targeted emails were sent to elicit more responses. Potential study participants (*N* = 729) were recruited to first complete an online survey via a Qualtrics link (NSGC, 2023). The number of potential participants was ascertained based on the number of 2023 NSGC professional status survey respondents who reported working in a clinical prenatal setting. A total of 35 prenatal GCs completed the survey. Upon completion of the survey, participants were given the option to enter a raffle for 10 $15 Amazon gift cards as a participation incentive. A total of 12 survey respondents agreed to participate in a semi‐structured qualitative interview about changes in their practice since the overturn and emotional well‐being/job satisfaction. The 12 participants practice in 10 unique states. Of note, one participant did not report the state in which she practices; however, she provided sufficient information about the changes to the laws in her state that allowed us to identify a state category for analysis. Participants were selected to receive an interview invite to capture the greatest diversity of states. They were split into the four state categories: (Stable Restrictive, SR (*N* = 2); Newly Restrictive, NR (*N* = 2); Unstable, US (*N* = 3); Stable Permissive, SP (*N* = 5)) (Figure 1). Participants received a $25 Amazon gift card to compensate them for their time.

Individual semi‐structured interviews were conducted to generate qualitative data through an inductive and deductive approach (Corbin & Strauss, 2008).

### 2.3 Data generation

The survey was distributed in January and February of 2023. It contained 22–27 questions (depending on skip logic) about abortion referral/coordination practices. A survey guide detailing relevant topics and specific questions was developed based on authors' experiences in the prenatal field and existing literature on the effects of abortion restrictions on prenatal genetic counseling practice (Cooney et al., 2017; Heuerman et al., 2022; Jayaraman et al., 2021; Koenig et al., 2019). Participants were asked about practices leading up to the Roe v. Wade overturn on June 24, 2022, and asked about those same practices after the overturn, specifically from June 24, 2022, through November 8, 2022, which was midterm Election Day that year. The survey contained questions regarding referral practices, including the overall number of referrals per month as well as the frequency of (1) referrals within the GC's own institution, (2) in‐state referrals, and (3) out‐of‐state referrals. The survey also asked about the distance to the nearest abortion clinic and how many days patients had to wait on average to have an abortion appointment. Lastly, the survey included questions regarding how often GCs helped with specific abortion coordination tasks such as mandatory state counseling and identifying supplemental funding for patients. Respondents were also asked for demographics including, but not limited to, the number of years practicing, institutional setting, and state in which they practice.

Separate interviews were conducted by the primary investigator in March and April of 2023. An interview guide to introduce topics and structure the conversation was developed based on researchers' experience in prenatal genetic counseling and existing literature on the effects of abortion restrictions on prenatal genetic counseling practice (Cooney et al., 2017; Heuerman et al., 2022; Jayaraman et al., 2021; Koenig et al., 2019). EH's professional background in reproductive health policy and public health also shaped topics of interest for the interview guide. The interview guide contained sections on five topics: (1) demographics; (2) preparation for the Roe v. Wade overturn; (3) counseling/practice changes since the Roe v. Wade overturn; (4) ability to fulfill job duties; and (5) job satisfaction/emotional well‐being. These topics were chosen to assess changes in prenatal genetic counseling practice after the overturn and to expand on topics examined in previous literature on the intersection of prenatal genetic counseling and abortion restrictions. The primary investigator completed two pilot interviews with GCs currently practicing in a clinical prenatal setting (one of whom was on the research team) to ensure that the interview guide was consistent with the desired length, content, and flow. At the start of each interview, the interviewer outlined the interview topics and addressed potential participant concerns. Consent was obtained before beginning the video and audio recording. The interviewer did not discuss her personal views on abortion or the impact of abortion restrictions on prenatal genetic counseling practice during the interviews so as not to influence responses. Audio transcripts with no personal identifiers were downloaded and edited to remove transcription errors and enhance clarity. Video recordings were downloaded to aid in transcript cleaning and deleted 150 days after interviews were completed. Interviews ranged from 23 to 54 min, with an average of 38.7 min per interview.

### 2.4 Analysis: quantitative data

Paired *t*‐tests were used to compare “before” responses to “after” responses, and one‐way ANOVA and Tukey tests were subsequently performed to compare differences in responses between state categories. Analysis was completed with the program R (Version 2022.12.0 + 353) (R Core Team, 2021; Vienna, Austria).

### 2.5 Analysis: coding and theme generation

Semi‐structured qualitative interviews were recorded and transcribed over Zoom (Version 5.17.11) in March and April 2023 (Zoom Video Communications; California, USA). Transcripts were analyzed using Dedoose (2021) (Version 9.0.17) qualitative data analysis software (SocioCultural Research Consultants LLC; California, USA). At least two research team members read and independently coded each transcript. We utilized reflexive thematic analysis to allow for flexibility in the coding process throughout analysis (Braun & Clarke, 2024). Five codes were developed based on existing literature and the results from the study survey, and six codes were generated in reading the first three initial transcripts. Themes were developed both deductively (using the interview guide based on existing literature and researchers' experiences in prenatal practice) and inductively (based on new information elicited in the interviews and elements of impact on clinical practice). The final step of theme generation was determining what themes were cross‐cutting versus specific to the state categories. The clinical prenatal experience of three of the four researchers was essential in the development and evaluation of codes and both state‐specific and cross‐cutting themes. The qualitative research experience of one researcher was used to guide this process. Discussion between all the researchers was used to determine when sufficient information power had been achieved (Malterud et al., 2016).

## 3 RESULTS

### 3.1 Participant survey demographics

A total of 35 GCs representing all four state categories responded to the survey (US *N* = 8, SR *N* = 5, NR *N* = 5, SP *N* = 17). Respondents had from 1 year to over 20 years of experience in prenatal genetic counseling. Respondents practiced in a variety of institutional settings, with the majority practicing in academic medical centers (*n* = 21) and public nonreligious (*n* = 7) institutions. The religiously affiliated institutions were all of Catholic denomination (*n* = 4). The majority of participants (*n* = 22) worked at institutions with comprehensive abortion care (dilation and curettage, dilation and evacuation, and induction of labor) prior to the Roe v. Wade overturn. Participants held a variety of professional (as opposed to personal) beliefs on abortion: 22 believed abortion should be an option throughout pregnancy, 11 believed it should be available up to a certain point, and two believed it should not be an option at all during pregnancy. Table 1 summarizes the demographics of the survey participants.

**TABLE 1:** Survey participant demographics.

State category	Years of experience	Institution type	Abortion procedures offered by institution before June 24, 2022	Abortion procedures offered by institution at the time of survey completion (February 2023)
Newly restrictive (*N* = 4)	1–3 (*n* = 1) 10–15 (*n* = 1) 15–20 (*n* = 1) 20+ (*N* = 1)	Academic (*n* = 3) Private nonreligious (*n* = 1)	None (*n* = 3) D&C, D&E, IOL (*n* = 1)	None (*n* = 3) D&C, D&E, IOL (*n* = 1)
Stable permissive (*N* = 15)	1–3 (*n* = 4) 3–5 (*n* = 3) 5–10 (*n* = 2) 10–15 (*n* = 2) 15–20 (*n* = 1) 20+ (*n* = 3)	Academic (*n* = 10) Private nonreligious (*n* = 1) Public nonreligious (*n* = 3) Private religious (*n* = 1)	None (*n* = 1) D&E, IOL (*n* = 1) D&C, D&E, IOL (*n* = 13)	None (*n* = 1) D&E, IOL (*n* = 1) D&C, D&E, IOL (*n* = 13)
Stable restrictive (*N* = 5)	1–3 (*n* = 2) 5–10 (*n* = 2) 15–20 (*n* = 1)	Academic (*n* = 4) Public religious (*n* = 1)	None (*n* = 3) D&C, IOL (*n* = 1) D&C, D&E, IOL (*n* = 1)	None (*n* = 5)
Unstable (*N* = 6)	1–3 (*n* = 2) 3–5 (*n* = 1) 5–10 (*n* = 1) 10–15 (*n* = 1) 20+ (*n* = 1)	Academic (*n* = 3) Public nonreligious (*n* = 1) Private religious (*n* = 1) Public religious (*n* = 1)	None (*n* = 3) D&C, D&E, IOL (*n* = 3)	None (*n* = 4) D&C, D&E, IOL (*n* = 1) No response (*n* = 1)
Participant did not report state (*N* = 5)	3–5 (*n* = 3) 10–15 (*n* = 2)	Academic (*n* = 1) Private nonreligious (*n* = 1) Public nonreligious (*n* = 3)	D&C, D&E (*n* = 1) D&C, D&E, IOL (*n* = 4)	D&C, D&E (*n* = 1) D&C, D&E, IOL (*n* = 4)

Abbreviations: D&C, dilation and curettage; D&E, dilation and evacuation; IOL, induction of labor.

### 3.2 Survey findings

Eliciting information from prenatal GCs on their abortion referral and coordination practices before versus after the Roe v. Wade overturn illuminated a variety of changes in the abortion access landscape.

#### 3.2.1 Distance to nearest abortion provider

Before the Roe v. Wade overturn 6% of GCs reported a distance of 100+ miles from their clinic to the nearest abortion clinic (*N* = 34). Comparison across the state categories illustrated differences in distance reported between GCs practicing in SR versus SP states (*p* = 0.006) as well as SR versus US states (*p* = 0.02). Analysis of the data from after the Roe v. Wade overturn found an increase in the average reported distance to the nearest abortion clinic, with a 26% increase in GCs reporting distances of 100+ miles to the nearest clinic (*p* = 0.002; *N* = 34). There were differences between SP states versus all other state categories after the Roe v. Wade overturn (SP/NR *p* = 0.01; SP/US *p* = 0.007), though the greatest difference in clinic distance was between SR versus SP states (*p* < 0.001). These findings are summarized in Table 2.

**TABLE 2:** Changes in abortion coordination and referral practices of prenatal GCs following the overturn of Roe v. Wade.

Abortion coordination/referral demographics and practices (*N* = 34)	Percentage of GCs reporting pre‐overturn	Post‐overturn	Percent changes	*p*‐Value
Distance of 100+ miles to nearest abortion provider (*N* = 34)	6% (*n* = 2)	33% (*n* = 11)	+26%	0.002
Wait time of 7+ days for abortion appointment (*N* = 30)	13% (*n* = 4)	57% (*n* = 17)	+44%	<0.001
Refer patients out‐of‐state “Always” (*N* = 33)	9% (*n* = 3)	30% (*n* = 10)	+21%	0.009

#### 3.2.2 Wait time for abortion appointment

Before the Roe v. Wade overturn, 87% of GCs surveyed reported that their patients waited 0–7 days to receive abortion care, while the remaining 13% of GCs reported wait times of 7–14 days (*N* = 30). There was a significant increase in the reported wait times after the Roe v. Wade overturn (*p* < 0.001), with a 44% increase in GCs reporting patients waiting 7+ days for an appointment after the overturn (*N* = 30). Of that 44%, 17% reported wait times of 14–21 days. Notably, there were no significant differences in reported wait times between state categories either before or after the overturn. This indicates that wait times were generally the same across state categories before the Roe v. Wade overturn and saw similar increases between all state categories after the overturn. These findings are summarized in Table 2.

#### 3.2.3 Frequency of out‐of‐state abortion referrals

Before the Roe v. Wade overturn, 9% of GCs reported that they often or always referred patients out‐of‐state (*N* = 33). State category comparisons showed a significant difference in the frequency of out‐of‐state referrals before the Roe v. Wade overturn between SP states versus SR states and SP states versus NR states (SP/SR *p* = 0.014; SP/NR *p* = 0.014). After the Roe v. Wade overturn, results showed an increase in out‐of‐state referrals (*p* = 0.009) accompanied by a decrease in in‐state referrals (*p* < 0.001). There was a 21% increase in GCs reporting they often or always refer out‐of‐state and a 26% increase in GCs reporting that they never referred in‐state. State comparisons found differences in the frequency of out‐of‐state referrals between SP states versus all other categories (SR *p* < 0.001, NR *p* = 0.005, US *p* = 0.008) after the Roe v. Wade overturn. The greatest differences between in‐ and out‐of‐state referral frequency were SR versus SP states (*p* < 0.001). These findings are summarized in Table 2.

### 3.3 Participant interview demographics

A total of 12 prenatal GCs were interviewed representing the four state categories (US *N* = 3; SR *N* = 2; NR *N* = 2; SP *N* = 5). Respondents had levels of experience in prenatal genetic counseling ranging from 1 year to over 20 years, with a median of 5–10 years of experience. Respondents practiced in a variety of institutional settings, with the majority practicing in academic medical centers and public nonreligious institutions. The majority of participants (*n* = 6) worked at institutions with comprehensive abortion care (dilation and curettage, dilation and evacuation, and induction of labor) or did not offer abortion care at all before the Roe v. Wade overturn (*n* = 5). The remaining participant worked at an institution that offered only dilation and evacuation and induction of labor before the overturn. Of the 12 participants, four had the professional belief that abortion should be an option up to a certain point in pregnancy, while the remaining eight believed it should be an option throughout the entire pregnancy. Table 3 summarizes the demographics of the interview participants.

**TABLE 3:** Interview participant demographics.

State category	Participant #	Years of experience	Institution type	Abortion procedures offered by institution before June 24, 2022	Abortion procedures offered by institution at the time of survey completion (February 2023)
Newly restrictive	1	10–15	Academic	None	None
	2	15–20	Academic	None	None
Stable permissive	3	1–3	Public nonreligious	D&C, D&E, IOL	D&C, D&E, IOL
	4	3–5	Private nonreligious	D&C, D&E, IOL	D&C, D&E, IOL
	5	3–5	Academic	D&C, D&E, IOL	D&C, D&E, IOL
	6	10–15	Academic	D&C, D&E, IOL	D&C, D&E, IOL
	7	20+	Academic	D&E, IOL	D&E, IOL
Stable restrictive	8	1–3	Academic	None	None
	9	5–10	Academic	None	None
Unstable	10	5–10	Public nonreligious	None	None
	11	10–15	Academic	D&C, D&E, IOL	None
	12	3–5	Public nonreligious	D&C, D&E, IOL	D&C, D&E, IOL

Abbreviations: D&C, dilation and curettage; D&E, dilation and evacuation; IOL, induction of labor.

### 3.4 Interview findings

In discussing the factors that were impacting prenatal genetic counseling practice after the Roe v. Wade overturn, the researchers generated three themes impacting all participants regardless of their state of practice: (1) GCs have altered their practice due to legal uncertainty and changing policies, including utilizing workarounds, altering documentation, and providing earlier and more detailed anticipatory guidance; (2) GCs have responded to changing policies by building their own resource networks and self‐educating in the absence of clear institutional guidance and a lack of legal resources; and (3) GCs experienced increased burden from their role in abortion coordination and care, at least in part due to demands of self‐education about the laws and regulations surrounding abortion (listed in Table 4).

**TABLE 4:** Cross‐cutting themes constructed from the interviews of the 12 study participants are listed in the table.

Cross‐cutting themes
GCs have altered their practice due to legal uncertainty and changing policies, including utilizing work arounds, altering documentation, and providing earlier and more detailed anticipatory guidance
2 GCs have responded to changing policies by building their own resource networks and self‐educating in the absence of clear institutional guidance and a lack of legal resources
3 GCs experienced increased burden from their role in abortion coordination and care, at least in part due to demands of self‐education and the laws and regulations surrounding abortion

### 3.5 Cross‐cutting themes

#### 3.5.1 Theme 1: GCs have altered their practice due to legal uncertainty and changing policies, including utilizing workarounds, altering documentation, and providing earlier and more detailed anticipatory guidance

Participants discussed different types of uncertainty they faced after the Roe v. Wade overturn, including uncertainty about what laws were in effect, uncertainty about the future, and uncertainty about what they as GCs were legally allowed to say or do based on laws.

Things are changing so much, like it's in every state's courts. It's on ballot initiatives during the election. It's being argued at the federal level, it's being argued at the state level, it's being argued at the local level, like a municipality here nearby the city that I live in was trying to pass like an abortion ban within their municipality that included people who live outside of their municipality aiding and abetting people who live inside of it. So then that raises questions for us… if we have a patient who lives there and we give them information, is that aiding and abetting something? And… what does that mean? So it's definitely been more challenging just because there's just been so much more going on at …literally every level whereas before Dobbs, yes, there were debates, and yes, there were discussions, but given the protections under Roe, …they didn't really go anywhere (Participant 10, Unstable)

GCs navigated this uncertainty by changing their conversations with patients and their workflows. Participants explained how they began accounting for uncertainty due to frequent changes in the laws in both their state and surrounding states. Some began including disclaimers about the status of laws and options for accessing abortion in their discussions with patients.

…any time the topic of termination was raised or discussed, we would inform patients that you know, there was not an option for elective termination in the state of [state], and we did have other options out of state, but again, which states were offering elective terminations was also changing as well. (Participant 11, Unstable)

Participants also discussed changes in the logistics for patient access to an abortion. For example, many participants noted increased wait times for abortion appointments as a new barrier to access for patients seeking abortion. Wait times in different locations fluctuate with changes in state laws, so some participants provided additional anticipatory guidance on the longer wait times and other logistics in their counseling sessions. Some advised patients to schedule an appointment before test results returned if they were at all considering abortion so that wait times would not push them outside of their state gestational age limit preventing local abortion care.

Yeah, I typically now will tell people to start thinking about making an [abortion] appointment…as soon as we're doing an amniocentesis, if that's something that they think that they might want to access, so we've noticed, especially here in the [region] that there are month, multiple‐week‐long wait lists for a lot of [clinics] (Participant 8, Stable Restrictive)

In addition to providing more anticipatory guidance, most GCs described workaround efforts aimed at supporting access to abortion in the wake of increased restrictions after the Roe v. Wade overturn. Some participants mentioned advising patients to complete mandatory waiting period paperwork if they were considering abortion to avoid extra wait time while trying to access timeline‐restricted care.

After Roe, I feel like we started to be a little bit more pushy about…hey, if you are at all thinking that you might want to end this pregnancy, …you might just want to sign this consent form now so that it's not…a bureaucratic hold‐up to…access services…I started to ask the providers that were allowed to legally consent patients with that form to…be pulled in more often. (Participant 3, Stable Permissive)

Some participants discussed changes they made to written documentation because of the Roe v. Wade overturn. The main reason for these changes was to protect themselves and/or their patients. Participants described using more vague language regarding abortion counseling and referrals in their documentation and some only included the minimum amount of information necessary regarding abortion decision‐making, such as “discussed pregnancy management options.”

So you just never know like a patient might end up moving to a different state and …you don't wanna unnecessarily make things difficult. And you know, who knows what's gonna happen…I would say that we're trying to be a little bit more vague in the documentation, but not so much where anyone's ever got to the extent where any of us have ever discussed it. (Participant 4, Stable Permissive)

Our legal team has said, we can talk about anything we want regarding termination. We can facilitate …referrals and stuff like that with no legal ramifications, but I think all of us are worried about …that being true right now. But seeing other states where that's no longer true, we're worried about just stigma or problems that could come up in the future. Like, what if a new law gets passed next month that then makes this illegal or puts us in, at risk or liability and is there…a retroactive component to that, could we get in trouble? Could our patients get in trouble if we documented that? (Participant 10, Unstable)

Some participants mentioned concerns about the legal implications for themselves with written documentation and described having conversations around abortion over the phone rather than including it in patient documentation.

I was comfortable and still am emailing patients with information because it's information that's available online…[but] at least some of our coworkers I know are just not comfortable even having that …written trail of information that can come back to them. (Participant 10, Unstable)

#### 3.5.2 Theme 2: GCs Have responded to changing policies by building their own resource networks and self‐educating in the absence of clear institutional guidance and a lack of legal resources

GCs described utilizing a network of resources to keep up with changing information in the wake of the overturn. Many relied on social media and reproductive rights advocacy groups such as Planned Parenthood to track updates. Participants also used their fellow GCs to learn about and discuss changes. A few participants had MFMs on their team who helped keep everyone up to date; notably, GCs who had this resource described these types of MFMs as providers who were already very vocal advocates on the topic.

I follow …people on social media, so Planned Parenthood and other advocacy groups on social media so that was helpful, my coworkers, so those at my institution, but then also other prenatal genetic counselors in our city and area, we would talk and have…e‐mail and text chains. We have…a group text message that whenever somebody would hear an update about something they would post because it is really hard to stay on top of things and there's not…a central place to go often, especially when we're thinking about all the surrounding states around us. So a lot of it was just word of mouth and discussing it with coworkers. (Participant 12, Unstable)

Institutional guidance, if available, was useful for GCs, although not everyone received clear or timely guidance from their institution. One participant described needing to repeatedly ask for legal interpretation of laws from their institution in order to understand legal implications. Legal resources were helpful to GCs, whether from their institution or an independent source, because they allowed them to better understand the laws in effect and what the laws meant for patient care. Some GCs who received quick and clear guidance described an increase in satisfaction with their institution after the Roe v. Wade overturn directly related to that support.

#### 3.5.3 Theme 3: GCs experienced increased burden from their role in abortion coordination and care, at least in part due to demands of self‐education of the laws

Participants named various roles GCs have in abortion coordination both before and after the Roe overturn. These roles included researching abortion laws, educating clinic staff on laws, and coordinating abortion care for patients. While these are roles GCs were responsible for before the overturn, the participants described how these roles have become more burdensome in the aftermath of the overturn. In line with the survey results, many participants discussed increased barriers to abortion access after the overturn, with wait times, gestational age restrictions, and distance to abortion clinics as the most mentioned barriers.

GCs described how it was necessary for them to keep up with laws in order to adequately discuss pregnancy management options with patients:

I pay more attention to evolving abortion laws than I do to new literature being published on scientific advances…because my job so frequently involves helping people make those kinds of decisions and making arrangements once the decisions are made…practically speaking, it's required for me to know that (Participant 6, Stable Permissive)

GCs discussed how they are now often the point person on their team keeping track of abortion laws. Additionally, GCs noted they were the point person due to clinic expectations, not because they had volunteered to fill the role. GCs explained the burden of tracking laws increased after the overturn as there were more frequent changes to laws in either the GC's own state, surrounding states, or both.

I'm really the person on our team that MFMs typically turn to for information about where they can refer patients and where they can still send people. So I just try to stay as up‐to‐date as possible just for patients' benefit. (Participant 8, Stable Restrictive)

I feel like I know the resources where I can go to access the information I would need to figure [out state laws], but honestly it's been pretty much impossible to keep up with every single state in the country and what's going on there [post‐overturn]. (Participant 8, Stable Restrictive)

Some participants also discussed increased frustration with members of their team and the expectation that the GCs be solely responsible for understanding the restrictions and options for patients. Because GCs are often in charge of researching and understanding the laws, they were also expected to educate other clinic staff on the laws and abortion care options, ranging from the maternal‐fetal medicine (MFM) physicians to nursing staff.

So there's a lot of well, just go talk to your genetic counselor, cause they know what's supposed to happen, which is fine…but there's definitely a few more instances where I've been frustrated with my coworkers and wanted to just be like, why don't you do it? Why do I have to always do this, which I didn't ever really have those feelings before [the overturn]. (Participant 10, Unstable)

In addition to familiarity with abortion restrictions, GCs also described how they are one of the primary team members responsible for coordinating abortion care for patients. GCs needed to know what care options were available in their own state and institution, if any. Oftentimes they also needed to know referral locations for out‐of‐state due to gestational age restrictions and other laws within their own state. Determining where abortion care was available for patients became increasingly challenging as state laws rapidly changed post‐overturn.

I kept having to update my paper that I was giving to patients… those were pretty specific conversations [about that state] because [the state's gestational age limit] kept going back and forth based on injunctions. That was a week by week and then having to make a call to clinics to find out, you know, what their current status was. (Participant 1, Newly Restrictive)

### 3.6 Comparison across state categories

We made several observations about state‐category‐specific differences on the impact of the Roe v. Wade overturn that provide additional insight into our results. Our analysis found that despite the abortion coordination role becoming more burdensome for all GCs, the number of states that GCs were tracking laws for varied by the participant state category. GCs working in Stable Permissive states appeared to be mainly focused on tracking laws within their own state. Conversely, GCs practicing in Unstable, Stable Restrictive, and Newly Restrictive states appeared to track surrounding states' laws in addition to their own state, given how often they refer patients out of state for abortion care. Similarly, while uncertainty was noted in some capacity for all GCs, the specific type of uncertainty seemed to vary by state category. Most GCs mentioned legislative uncertainty—the uncertainty around what laws were in effect and what the laws meant—but GCs in Stable Permissive states brought up uncertainty about what laws may come into play in the future more often than GCs in the other categories from our sample. Additionally, GCs in restrictive states (both Stable and Newly) brought up uncertainty about the logistics of abortion care options more than Stable Permissive and Unstable GCs in our sample. These observations beg the question of what state‐category‐specific changes to clinical care have been made, suggesting the importance of further study to determine state‐specific themes.

Notably, while GCs in Stable Restrictive states made adaptations to their counseling after the Roe v. Wade overturn, they had already made significant changes to their practice due to total or near‐total bans on abortion that were in effect in their states before the overturn. The GCs in this category explained that they were more impacted by these previous bans than the Roe v. Wade overturn.

## 4 DISCUSSION

The overturning of Roe v. Wade in 2022 was an unprecedented nationwide change in abortion access policy in the United States. In the absence of the federal legal standard establishing a constitutional right to abortion up to viability (considered to be between 20 and 25 weeks and 6 days by ACOG), state legislatures can now enact significantly more restrictive gestational age bans than were possible under Roe v. Wade. A novel product of the Roe v. Wade overturn is the instability of abortion laws in states where extremely restrictive gestational age bans have been continually cycling in and out of effect due to lawsuits. This study provides insight into how prenatal GCs have adapted their practice to the increased restrictions and uncertainty resulting from the lack of federal standard due to the Roe v. Wade overturn. Interviewees described various barriers to abortion access for their patients, with the majority listing gestational age restrictions and wait times as most contributory. Gestational age bans were previously described as an obstacle for patients before the Roe v. Wade overturn, but significant wait times due to clinic volume are a newfound barrier. The Guttmacher Institute's Monthly Abortion Provision Study has recorded an 11.1% increase in clinician‐provided abortions in states without a total ban since 2020, indicating an increase in patients traveling to clinics in states without bans to receive care (Maddow‐Zimet et al., 2024). Most interview participants discussed that these increased wait times were a barrier to abortion care for patients, regardless of their state category. The combination of frequently changing laws and increasing restrictions across the country has made genetic counseling roles in abortion coordination more laborious and has changed how GCs counsel their patients regarding abortion options. GCs expressed concern over what further restrictions may be passed in the future. With the looming possibility of a restrictive nationwide gestational age ban, this study allows a window into some possible effects of a nationwide ban by examining prenatal genetic counseling practices in different states with a variety of types of abortion restrictions.

Many other types of abortion restrictions have been previously shown to impact prenatal genetic counseling practice, such as mandatory waiting periods, procedural restrictions (i.e., restricting which type of providers can perform abortions), and certain insurance coverage restrictions (Jayaraman et al., 2021; Koenig et al., 2019). While gestational age bans were a major discussion point brought up by GCs in interviews given the passage of harsher age restrictions in the absence of Roe, other state‐level restrictions that impact prenatal genetic counseling are still being passed. According to the Guttmacher Institute, 675 state‐level provisions that would restrict access to abortion care were introduced in 2023 as compared to only 563 abortion restrictions introduced in 2022 (Guttmacher Institute, 2022a, 2022b, 2022c, 2023a, 2023b). The layering of additional types of restrictions on top of gestational age restrictions further complicates abortion coordination for prenatal GCs and access for patients.

### 4.1 Changes in abortion care access

Interestingly, the majority of survey and interview participants did not report changes in abortion procedures offered at their institution before versus after the overturn of Roe v. Wade. Participants who reported changes in offered procedures practiced in Stable Restrictive and Unstable states. Despite this, there were significant changes in access indicated by increases in distance to the nearest abortion clinic, wait times, and frequency of out‐of‐state referrals. This indicates that there are many factors contributing to abortion access outside of simply the available procedures at a specific institution. Several interviewees discussed that while their institution had not offered abortion care at any point, changes in what nearby institutions and standalone clinics could offer impacted their patients. Additionally, our survey participants were a small portion of the total prenatal GCs in the country; other GCs who did not participate may have experienced changes to their institutions' offerings. Results of the survey were similar to those reported by Rader et al. (2022), who described the increase in estimated average drive time to an abortion appointment in the United States from 27.8 min pre‐Roe overturn to 100.4 min in September 2022 post‐Roe overturn. Our survey findings highlighted existing differences in abortion access between state categories, which were further exacerbated by the Roe v. Wade overturn. These differences in access could increase health disparities due to the increased burden of travel and related costs for individuals residing in states with total and near‐total bans.

### 4.2 Recommendations

The results of this study highlight the need for additional guidance and support from professional organizations and institutions for prenatal GCs. National and state professional organizations should coordinate and provide resources to assist prenatal GCs in tracking laws and abortion care options. This could include resources such as town halls for prenatal providers to provide updates on the status of their practice, a volunteer‐run live resource list with weekly updates, and help connecting GCs with policy organizations for additional support. Institutions should provide staff early, proactive legal support to clarify and interpret laws so providers are clear on what care they can and cannot provide to patients. The primary focus of this legal guidance should be supporting providers rather than simply protecting the institution itself.

### 4.3 Prenatal genetic counselors' scope of practice

As prenatal GCs face increased logistical burden in the wake of the Roe v. Wade overturn and growing restrictions on abortion access for patients, it begs the question of just how much the roles of abortion coordinator and legal navigator truly fall within their scope of practice. As the landscape of abortion restrictions becomes increasingly complex, it will be important to consider how the landscape pushes the genetic counseling scope to evolve. There is not an individualized scope of practice for each subspecialty, but one broad scope of practice for all of genetic counseling; however, prenatal GCs have been expected to take on more specialized roles in abortion coordination. Some prenatal GCs may take on the role of abortion coordinator/legal navigator because it is simply expected of them. Others may take it on because it is within a GC's scope to discuss pregnancy management options and they feel they need to research and understand the laws to adequately discuss options with patients (NSGC, 2024). GCs have no legal training, and as abortion counseling has become so complex it may be more appropriate for institutions to utilize dedicated professionals who are experts in the legal and/or logistical details of pregnancy management to take this role out of the hands of GCs.

### 4.4 Limitations and future studies

While we were unable to draw conclusions about differences between state categories at the time of this study, our analysis indicates that it will be crucial to explore state‐specific issues in future studies. Given that this study was conducted in the early months after the Roe v. Wade overturn, only a limited number of states were categorized as Unstable or Newly Restrictive, therefore limiting the possible number of participants from each state category. Both Stable Restrictive interviewees were from the same state, and two of the three Unstable interviewees came from the same state. Follow‐up studies should aim to have more overall participants and as many from each state category as possible to gather more robust state category comparisons.

The vast majority of survey respondents and all interview participants felt abortion should be an option for patients; thus, the study did not capture a large variety of viewpoints. GCs who chose to participate in the study may have been more eager to share opinions due to their perceptions of the negative impact of the overturn on their practice. Another limitation is possible confirmation bias from the researchers, though the researchers aimed to be impartial in analysis and interpretation. The four researchers hold the professional belief that abortion should be an option for pregnant patients and worked to be cognizant of the impact of this perspective in all stages of the study.

This study captured an early time point after the overturning of Roe v. Wade and the landscape of abortion access has continued to change since the data was captured in early 2023. Additionally, the study survey only had 35 total participants (4.8% of prenatal GCs in the country, *N* = 729) (NSGC, 2023). Completing another time point would aid in understanding how practice has continued to change since the initial survey and interviews were completed. Seeing as more states fall under the Unstable category now than at the time of the original study, it would be possible to gather information from more individuals across different Unstable states, possibly leading to more generalizable results. Participants described perceived impacts on patients, but the study did not examine impacts from the patient perspective. Future studies should assess impacts of the overturn on prenatal genetic counseling from the patient perspective to gain a more accurate understanding. Another area of focus for future studies is the impact of certain types of bans, specifically “bounty hunter bans” and “reason bans.” Bounty hunter bans include language that allows and incentivizes citizens to report anyone they suspect to be involved in aiding and abetting abortion; reason bans restrict individuals from seeking abortion for a specific reason such as a fetal genetic diagnosis. Several of our participants discussed the effects of these bans on patient care as well as the patient–provider relationship. Further exploration of their impacts would be useful in determining how to support patients and providers in states with these restrictions. Finally, further studies should examine the mental and emotional impact of the Roe v. Wade overturn on prenatal GCs. Our study only gathered a small amount of data on this topic in the early months after the Roe v. Wade overturn; future studies examining long‐term impacts would be a valuable measure of prenatal GCs' job satisfaction, retention in the specialty, and burnout as a result of the Roe v. Wade overturn.

## 5 CONCLUSIONS

The Dobbs decision has negatively impacted prenatal genetic counseling practice across the United States. Prenatal GC roles in abortion coordination have become more burdensome since Roe was overturned due to frequent changes in legislation and the decreasing availability of abortion in some locations. Prenatal GCs have worked to find methods of supporting patient choice and access to abortion, but there is a need for further guidance and support as GCs navigate the new, uncertain landscape.

## AUTHOR CONTRIBUTIONS

Elizabeth J. C. Hart conceptualized the study design and contributed to data collection, analysis, interpretation, and writing of the manuscript. Beverly M. Yashar, R. Beth Dugan, and Anne C. Heuerman contributed to conceptualizing the study design, analysis, interpretation, and revising the manuscript. Authors Elizabeth J. C. Hart and Beverly M. Yashar confirm that they had full access to all the data in the study and take responsibility for the integrity of the data and the accuracy of the data analysis. All of the authors gave final approval of this version to be published and agree to be accountable for all aspects of the work in ensuring that questions related to the accuracy or integrity of any part of the work are appropriately investigated and resolved.

## CONFLICT OF INTEREST STATEMENT

The authors, Elizabeth J. C. Hart, Beverly M. Yashar, R. Beth Dugan, and Anne C. Heuerman, declare that they have no conflicts of interest to disclose.

## ETHICS STATEMENT

Animal studies: No nonhuman animal studies were carried out by the authors for this article.

## Supporting information


Data S1:

Data S2:

Data S3:

## Data Availability

The institutional review board‐approved research protocol requires that survey responses and interview transcripts remain confidential and on file with study personnel. Please direct any queries about the data to Elizabeth Hart primary (ehart9@iuhealth.org).
