# Supplementary material for: Before and after: The impact of the Roe v. Wade overturn on prenatal genetic counseling practice
Source: J Genet Couns. 2025 Aug 5;34(4):e70088. doi: 10.1002/jgc4.70088 (PMC12323291; doi:10.1002/jgc4.70088)
Supplement: Supplementary file 1 — Data S1: [file JGC4-34-0-s001.docx]

**R Code:**

##State comparisons##

##Set Working Directory state comparisons##

setwd("/Users/lizhart/Desktop/Research Thesis/Data and Analysis")

getwd()

install.packages(c("ggplot2", "ggpubr", "tidyverse", "broom", "AICcmodavg"))

library(ggplot2)

library(ggpubr)

library(tidyverse)

library(broom)

library(AICcmodavg)

##Reading in data from CSV##

statedata=read.csv("statedata.csv",na="NA",header = TRUE,colClasses = c("factor", "numeric", "numeric", "numeric","numeric","numeric","numeric","numeric","numeric","numeric","numeric","numeric","numeric","numeric","numeric","numeric","numeric","numeric","numeric","numeric","numeric","numeric","numeric","numeric","numeric","numeric","numeric","numeric"))

summary(statedata)

#number of referrals per month#

breferralanova<-aov(b_referral ~ statecat, data = statedata)

summary(breferralanova)

areferralanova<-aov(a_referral ~ statecat, data = statedata)

summary(areferralanova)

tukeybreferral<-TukeyHSD(breferralanova)

tukeyareferral<-TukeyHSD(areferralanova)

tukeybreferral

tukeyareferral

#nearest clinic distance#

bdistanceanova<-aov(b_distance ~ statecat, data = statedata)

summary(bdistanceanova)

adistanceanova<-aov(a_distance ~ statecat, data = statedata)

summary(adistanceanova)

tukeybdistance<-TukeyHSD(bdistanceanova)

tukeyadistance<-TukeyHSD(adistanceanova)

tukeybdistance

tukeyadistance

#within institution referrals#

bwithinreferralanova<-aov(b_in_instate ~ statecat, data = statedata)

summary(bwithinreferralanova)

awithinreferralanova<-aov(a_in_instate ~ statecat, data = statedata)

summary(awithinreferralanova)

tukeybwithinreferralanova<-TukeyHSD(bwithinreferralanova)

tukeyawithinreferralanova<-TukeyHSD(awithinreferralanova)

tukeybwithinreferralanova

tukeyawithinreferralanova

#in state referrals#

binstatereferralanova<-aov(b_instate ~ statecat, data = statedata)

summary(binstatereferralanova)

ainstatereferralanova<-aov(a_instate ~ statecat, data = statedata)

summary(ainstatereferralanova)

tukeybinstatereferralanova<-TukeyHSD(binstatereferralanova)

tukeyainstatereferralanova<-TukeyHSD(ainstatereferralanova)

tukeybinstatereferralanova

tukeyainstatereferralanova

#out of state referrals#

boutstatereferralanova<-aov(b_outstate ~ statecat, data = statedata)

summary(boutstatereferralanova)

aoutstatereferralanova<-aov(a_outstate ~ statecat, data = statedata)

summary(aoutstatereferralanova)

tukeyboutstatereferralanova<-TukeyHSD(boutstatereferralanova)

tukeyaoutstatereferralanova<-TukeyHSD(aoutstatereferralanova)

tukeyboutstatereferralanova

tukeyaoutstatereferralanova

#wait time#

bwaittimeanova<-aov(b_waittime ~ statecat, data = statedata)

summary(bwaittimeanova)

awaittimeanova<-aov(a_waittime ~ statecat, data = statedata)

summary(awaittimeanova)

tukeybwaittimeanova<-TukeyHSD(bwaittimeanova)

tukeyawaittimeanova<-TukeyHSD(awaittimeanova)

tukeybwaittimeanova

tukeyawaittimeanova

**R output:**

> summary(statedata)

statecat b_referral b_distance b_in_instate b_instate b_outstate b_waittime

1: 5 Min. :1.000 Min. :1.000 Min. :1.000 Min. :1.000 Min. :1.000 Min. :1.000

2: 5 1st Qu.:2.000 1st Qu.:1.000 1st Qu.:1.000 1st Qu.:3.000 1st Qu.:2.000 1st Qu.:1.000

3: 8 Median :2.000 Median :1.000 Median :3.000 Median :4.000 Median :3.000 Median :1.000

4:17 Mean :2.257 Mean :1.543 Mean :2.829 Mean :3.371 Mean :2.714 Mean :1.147

3rd Qu.:3.000 3rd Qu.:1.000 3rd Qu.:4.000 3rd Qu.:4.000 3rd Qu.:3.000 3rd Qu.:1.000

Max. :3.000 Max. :6.000 Max. :5.000 Max. :5.000 Max. :5.000 Max. :2.000

NA's :1

b_counsel b_us b_consent b_fetalrem b_suppfund b_insurancecov b_insurancerev

Min. :1.000 Min. :1.000 Min. :1.000 Min. :1.000 Min. :1.0 Min. :1.0 Min. :1.000

1st Qu.:1.000 1st Qu.:1.000 1st Qu.:1.000 1st Qu.:1.000 1st Qu.:1.0 1st Qu.:1.0 1st Qu.:1.000

Median :1.000 Median :1.000 Median :1.000 Median :1.000 Median :2.0 Median :2.0 Median :2.000

Mean :2.143 Mean :1.971 Mean :1.714 Mean :1.657 Mean :2.2 Mean :2.6 Mean :1.571

3rd Qu.:4.000 3rd Qu.:3.000 3rd Qu.:1.500 3rd Qu.:2.000 3rd Qu.:3.0 3rd Qu.:4.0 3rd Qu.:2.000

Max. :5.000 Max. :5.000 Max. :5.000 Max. :5.000 Max. :5.0 Max. :5.0 Max. :3.000

a_referral a_distance a_in_instate a_instate a_outstate a_waittime a_counsel

Min. :1.000 Min. :1.000 Min. :1.000 Min. :1.000 Min. :1.000 Min. :1.000 Min. :1.000

1st Qu.:2.000 1st Qu.:1.000 1st Qu.:1.000 1st Qu.:1.000 1st Qu.:2.000 1st Qu.:1.000 1st Qu.:1.000

Median :2.000 Median :1.000 Median :3.000 Median :3.000 Median :3.000 Median :2.000 Median :1.000

Mean :2.171 Mean :2.588 Mean :2.629 Mean :2.543 Mean :3.182 Mean :1.742 Mean :2.029

3rd Qu.:3.000 3rd Qu.:5.000 3rd Qu.:4.000 3rd Qu.:4.000 3rd Qu.:5.000 3rd Qu.:2.000 3rd Qu.:3.500

Max. :4.000 Max. :6.000 Max. :5.000 Max. :4.000 Max. :5.000 Max. :3.000 Max. :5.000

NA's :1 NA's :2 NA's :4

a_us a_consent a_fetalrem a_suppfund a_insurancecov a_insurancerev stance

Min. :1.000 Min. :1.000 Min. :1.000 Min. :1.000 Min. :1.0 Min. :1.000 Min. :1.000

1st Qu.:1.000 1st Qu.:1.000 1st Qu.:1.000 1st Qu.:1.000 1st Qu.:1.0 1st Qu.:1.000 1st Qu.:1.000

Median :1.000 Median :1.000 Median :1.000 Median :2.000 Median :2.0 Median :1.000 Median :1.000

Mean :1.943 Mean :1.829 Mean :1.686 Mean :2.114 Mean :2.4 Mean :1.514 Mean :1.486

3rd Qu.:3.500 3rd Qu.:2.000 3rd Qu.:2.000 3rd Qu.:3.000 3rd Qu.:4.0 3rd Qu.:2.000 3rd Qu.:2.000

Max. :5.000 Max. :5.000 Max. :5.000 Max. :5.000 Max. :5.0 Max. :3.000 Max. :4.000

>

> #number of referrals per month#

>

> breferralanova<-aov(b_referral ~ statecat, data = statedata)

> summary(breferralanova)

Df Sum Sq Mean Sq F value Pr(>F)

statecat 3 1.281 0.4271 1.408 0.259

Residuals 31 9.404 0.3034

>

> areferralanova<-aov(a_referral ~ statecat, data = statedata)

> summary(areferralanova)

Df Sum Sq Mean Sq F value Pr(>F)

statecat 3 4.261 1.42 3.464 0.028 *

Residuals 31 12.710 0.41

---

Signif. codes: 0 ‘***’ 0.001 ‘**’ 0.01 ‘*’ 0.05 ‘.’ 0.1 ‘ ’ 1

>

> tukeybreferral<-TukeyHSD(breferralanova)

>

> tukeyareferral<-TukeyHSD(areferralanova)

>

> tukeybreferral

Tukey multiple comparisons of means

95% family-wise confidence level

Fit: aov(formula = b_referral ~ statecat, data = statedata)

$statecat

diff lwr upr p adj

2-1 -0.60000000 -1.5454451 0.3454451 0.3296377

3-1 -0.02500000 -0.8772127 0.8272127 0.9998149

4-1 -0.10588235 -0.8663981 0.6546334 0.9812798

3-2 0.57500000 -0.2772127 1.4272127 0.2784010

4-2 0.49411765 -0.2663981 1.2546334 0.3097203

4-3 -0.08088235 -0.7218069 0.5600422 0.9859140

>

> tukeyareferral

Tukey multiple comparisons of means

95% family-wise confidence level

Fit: aov(formula = a_referral ~ statecat, data = statedata)

$statecat

diff lwr upr p adj

2-1 4.440892e-16 -1.09912811 1.099128 1.0000000

3-1 7.500000e-02 -0.91574069 1.065741 0.9968650

4-1 7.294118e-01 -0.15472652 1.613550 0.1351091

3-2 7.500000e-02 -0.91574069 1.065741 0.9968650

4-2 7.294118e-01 -0.15472652 1.613550 0.1351091

4-3 6.544118e-01 -0.09069562 1.399519 0.1014039

>

> #nearest clinic distance#

>

> bdistanceanova<-aov(b_distance ~ statecat, data = statedata)

> summary(bdistanceanova)

Df Sum Sq Mean Sq F value Pr(>F)

statecat 3 17.25 5.749 5.329 0.00444 **

Residuals 31 33.44 1.079

---

Signif. codes: 0 ‘***’ 0.001 ‘**’ 0.01 ‘*’ 0.05 ‘.’ 0.1 ‘ ’ 1

>

> adistanceanova<-aov(a_distance ~ statecat, data = statedata)

> summary(adistanceanova)

Df Sum Sq Mean Sq F value Pr(>F)

statecat 3 91.02 30.340 15.37 3.06e-06 ***

Residuals 30 59.21 1.974

---

Signif. codes: 0 ‘***’ 0.001 ‘**’ 0.01 ‘*’ 0.05 ‘.’ 0.1 ‘ ’ 1

1 observation deleted due to missingness

>

> tukeybdistance<-TukeyHSD(bdistanceanova)

>

> tukeyadistance<-TukeyHSD(adistanceanova)

>

> tukeybdistance

Tukey multiple comparisons of means

95% family-wise confidence level

Fit: aov(formula = b_distance ~ statecat, data = statedata)

$statecat

diff lwr upr p adj

2-1 -0.800000000 -2.582796 0.9827964 0.6204448

3-1 -1.875000000 -3.481991 -0.2680091 0.0172208

4-1 -1.882352941 -3.316434 -0.4482721 0.0063166

3-2 -1.075000000 -2.681991 0.5319909 0.2854311

4-2 -1.082352941 -2.516434 0.3517279 0.1926488

4-3 -0.007352941 -1.215924 1.2012184 0.9999983

>

> tukeyadistance

Tukey multiple comparisons of means

95% family-wise confidence level

Fit: aov(formula = a_distance ~ statecat, data = statedata)

$statecat

diff lwr upr p adj

2-1 -1.850000 -4.412637 0.7126370 0.2244301

3-1 -2.350000 -4.527823 -0.1721773 0.0306079

4-1 -4.482353 -6.425845 -2.5388611 0.0000038

3-2 -0.500000 -2.839357 1.8393568 0.9369741

4-2 -2.632353 -4.755285 -0.5094212 0.0105689

4-3 -2.132353 -3.770230 -0.4944756 0.0068872

>

> #within institution referrals#

>

> bwithinreferralanova<-aov(b_in_instate ~ statecat, data = statedata)

> summary(bwithinreferralanova)

Df Sum Sq Mean Sq F value Pr(>F)

statecat 3 29.19 9.73 8.928 0.000206 ***

Residuals 31 33.78 1.09

---

Signif. codes: 0 ‘***’ 0.001 ‘**’ 0.01 ‘*’ 0.05 ‘.’ 0.1 ‘ ’ 1

>

> awithinreferralanova<-aov(a_in_instate ~ statecat, data = statedata)

> summary(awithinreferralanova)

Df Sum Sq Mean Sq F value Pr(>F)

statecat 3 49.00 16.334 16.24 1.58e-06 ***

Residuals 31 31.17 1.006

---

Signif. codes: 0 ‘***’ 0.001 ‘**’ 0.01 ‘*’ 0.05 ‘.’ 0.1 ‘ ’ 1

>

> tukeybwithinreferralanova<-TukeyHSD(bwithinreferralanova)

>

> tukeyawithinreferralanova<-TukeyHSD(awithinreferralanova)

>

> tukeybwithinreferralanova

Tukey multiple comparisons of means

95% family-wise confidence level

Fit: aov(formula = b_in_instate ~ statecat, data = statedata)

$statecat

diff lwr upr p adj

2-1 0.2000000 -1.5919070 1.991907 0.9901532

3-1 1.3500000 -0.2652031 2.965203 0.1276203

4-1 2.2470588 0.8056494 3.688468 0.0010471

3-2 1.1500000 -0.4652031 2.765203 0.2357001

4-2 2.0470588 0.6056494 3.488468 0.0029188

4-3 0.8970588 -0.3176887 2.111806 0.2082847

>

> tukeyawithinreferralanova

Tukey multiple comparisons of means

95% family-wise confidence level

Fit: aov(formula = a_in_instate ~ statecat, data = statedata)

$statecat

diff lwr upr p adj

2-1 0.600000 -1.1212463 2.321246 0.7804026

3-1 0.750000 -0.8015104 2.301510 0.5624846

4-1 2.823529 1.4389595 4.208099 0.0000265

3-2 0.150000 -1.4015104 1.701510 0.9935402

4-2 2.223529 0.8389595 3.608099 0.0007357

4-3 2.073529 0.9066833 3.240375 0.0002001

>

> #in state referrals#

>

> binstatereferralanova<-aov(b_instate ~ statecat, data = statedata)

> summary(binstatereferralanova)

Df Sum Sq Mean Sq F value Pr(>F)

statecat 3 9.967 3.322 4.255 0.0125 *

Residuals 31 24.204 0.781

---

Signif. codes: 0 ‘***’ 0.001 ‘**’ 0.01 ‘*’ 0.05 ‘.’ 0.1 ‘ ’ 1

>

> ainstatereferralanova<-aov(a_instate ~ statecat, data = statedata)

> summary(ainstatereferralanova)

Df Sum Sq Mean Sq F value Pr(>F)

statecat 3 40.89 13.63 35.83 3.39e-10 ***

Residuals 31 11.79 0.38

---

Signif. codes: 0 ‘***’ 0.001 ‘**’ 0.01 ‘*’ 0.05 ‘.’ 0.1 ‘ ’ 1

>

> tukeybinstatereferralanova<-TukeyHSD(binstatereferralanova)

>

> tukeyainstatereferralanova<-TukeyHSD(ainstatereferralanova)

>

> tukeybinstatereferralanova

Tukey multiple comparisons of means

95% family-wise confidence level

Fit: aov(formula = b_instate ~ statecat, data = statedata)

$statecat

diff lwr upr p adj

2-1 -0.80000000 -2.31676319 0.7167632 0.4899424

3-1 0.62500000 -0.74219186 1.9921919 0.6063884

4-1 0.70588235 -0.51420145 1.9259662 0.4097996

3-2 1.42500000 0.05780814 2.7921919 0.0385301

4-2 1.50588235 0.28579855 2.7259662 0.0109035

4-3 0.08088235 -0.94734306 1.1091078 0.9964874

>

> tukeyainstatereferralanova

Tukey multiple comparisons of means

95% family-wise confidence level

Fit: aov(formula = a_instate ~ statecat, data = statedata)

$statecat

diff lwr upr p adj

2-1 0.200000 -0.85870792 1.258708 0.9554363

3-1 1.125000 0.17069357 2.079306 0.0158822

4-1 2.588235 1.73661099 3.439860 0.0000000

3-2 0.925000 -0.02930643 1.879306 0.0601406

4-2 2.388235 1.53661099 3.239860 0.0000001

4-3 1.463235 0.74552906 2.180942 0.0000266

>

> #out of state referrals#

>

> boutstatereferralanova<-aov(b_outstate ~ statecat, data = statedata)

> summary(boutstatereferralanova)

Df Sum Sq Mean Sq F value Pr(>F)

statecat 3 14.10 4.701 5.82 0.00282 **

Residuals 31 25.04 0.808

---

Signif. codes: 0 ‘***’ 0.001 ‘**’ 0.01 ‘*’ 0.05 ‘.’ 0.1 ‘ ’ 1

>

> aoutstatereferralanova<-aov(a_outstate ~ statecat, data = statedata)

> summary(aoutstatereferralanova)

Df Sum Sq Mean Sq F value Pr(>F)

statecat 3 40.10 13.367 12.58 1.91e-05 ***

Residuals 29 30.81 1.062

---

Signif. codes: 0 ‘***’ 0.001 ‘**’ 0.01 ‘*’ 0.05 ‘.’ 0.1 ‘ ’ 1

2 observations deleted due to missingness

>

> tukeyboutstatereferralanova<-TukeyHSD(boutstatereferralanova)

>

> tukeyaoutstatereferralanova<-TukeyHSD(aoutstatereferralanova)

>

> tukeyboutstatereferralanova

Tukey multiple comparisons of means

95% family-wise confidence level

Fit: aov(formula = b_outstate ~ statecat, data = statedata)

$statecat

diff lwr upr p adj

2-1 4.440892e-16 -1.542713 1.5427129 1.0000000

3-1 -7.250000e-01 -2.115583 0.6655827 0.4998048

4-1 -1.482353e+00 -2.723311 -0.2413952 0.0142927

3-2 -7.250000e-01 -2.115583 0.6655827 0.4998048

4-2 -1.482353e+00 -2.723311 -0.2413952 0.0142927

4-3 -7.573529e-01 -1.803170 0.2884640 0.2227831

>

> tukeyaoutstatereferralanova

Tukey multiple comparisons of means

95% family-wise confidence level

Fit: aov(formula = a_outstate ~ statecat, data = statedata)

$statecat

diff lwr upr p adj

2-1 -1.000000 -2.776043 0.7760425 0.4310173

3-1 -1.375000 -2.975903 0.2259031 0.1122131

4-1 -2.933333 -4.383466 -1.4832007 0.0000349

3-2 -0.375000 -1.975903 1.2259031 0.9187700

4-2 -1.933333 -3.383466 -0.4832007 0.0056036

4-3 -1.558333 -2.787743 -0.3289236 0.0088314

>

> #wait time#

>

> bwaittimeanova<-aov(b_waittime ~ statecat, data = statedata)

> summary(bwaittimeanova)

Df Sum Sq Mean Sq F value Pr(>F)

statecat 3 0.406 0.1353 1.052 0.384

Residuals 30 3.859 0.1286

1 observation deleted due to missingness

>

> awaittimeanova<-aov(a_waittime ~ statecat, data = statedata)

> summary(awaittimeanova)

Df Sum Sq Mean Sq F value Pr(>F)

statecat 3 2.906 0.9687 2.007 0.137

Residuals 27 13.029 0.4826

4 observations deleted due to missingness

>

> tukeybwaittimeanova<-TukeyHSD(bwaittimeanova)

>

> tukeyawaittimeanova<-TukeyHSD(awaittimeanova)

>

> tukeybwaittimeanova

Tukey multiple comparisons of means

95% family-wise confidence level

Fit: aov(formula = b_waittime ~ statecat, data = statedata)

$statecat

diff lwr upr p adj

2-1 -2.000000e-01 -0.8541838 0.4541838 0.8391789

3-1 -2.000000e-01 -0.7559493 0.3559493 0.7627787

4-1 3.529412e-02 -0.4608358 0.5314240 0.9973767

3-2 -2.220446e-16 -0.5971853 0.5971853 1.0000000

4-2 2.352941e-01 -0.3066428 0.7772310 0.6435026

4-3 2.352941e-01 -0.1828193 0.6534075 0.4327575

>

> tukeyawaittimeanova

Tukey multiple comparisons of means

95% family-wise confidence level

Fit: aov(formula = a_waittime ~ statecat, data = statedata)

$statecat

diff lwr upr p adj

2-1 -1.000000e+00 -2.3442223 0.3442223 0.2001630

3-1 -1.000000e+00 -2.2271014 0.2271014 0.1406776

4-1 -7.941176e-01 -1.8505490 0.2623137 0.1928441

3-2 8.881784e-16 -1.2271014 1.2271014 1.0000000

4-2 2.058824e-01 -0.8505490 1.2623137 0.9501745

4-3 2.058824e-01 -0.6968311 1.1085958 0.9234269
