# Supplementary material for: Before and after: The impact of the Roe v. Wade overturn on prenatal genetic counseling practice
Source: J Genet Couns. 2025 Aug 5;34(4):e70088. doi: 10.1002/jgc4.70088 (PMC12323291; doi:10.1002/jgc4.70088)
Supplement: Supplementary file 2 — Data S2: [file JGC4-34-0-s003.docx]

**Informed Consent**

Thank you for your willingness to participate in this interview. This interview should take 30-60 minutes, is now still a good time to talk?

This interview will be audio recorded. The purpose of recording is to ensure the accuracy of the auto-generated transcript. Do I have your permission to record you? START RECORDING.

The Human Subject Incentives Program at the University of Michigan will coordinate the distribution of these gift cards. I anticipate that they should be sent out (Month 2023).

We will protect your identity by deidentifying the data and breaking the link between the study survey and the interview recruitment survey. We will not list your state of practice in our write-up of the data collected; states will be put into categories such as “restrictive” and the states in each category will not be listed either in an effort to maintain anonymity for counselors from states with fewer prenatal genetic counselors.

As an overview of what I am going to ask about today, we will start by discussing your background and your workplace a little more. Then, we will discuss the overturn of *Roe v. Wade* and changes you’ve made to your counseling. Last, we will talk about your job satisfaction and emotional wellbeing in the wake of the overturn.

We are conducting this research because we want to understand how the *Roe v. Wade* overturn has changed prenatal genetic counseling practices and its effect on prenatal genetic counselors. Because that is our goal, there is no right or wrong answer to any of these questions. You should know that everything you share in this interview will be kept confidential. You also may choose not to answer any questions or stop the interview at any time. If you need to take a break at any time, please let me know. I will also pause about halfway through to see if you need a break for water or to use the bathroom or anything else.

Do you have any questions before we begin the interview?

**Section 1: General Background**

To start, I’d like to get a little more background on your time working as a prenatal genetic counselor and about your workplace.

- *How long have you practiced in the prenatal setting?*
- *How did you come to be in prenatal as your specialty?*
- *How many other prenatal counselors do you work with in your team, if any?*
- *Your survey indicated you are X level of familiar with abortion laws in your state, can you tell me more about why you chose that response?*
- *Can you tell me more about your general stance on abortion and abortion restriction?*
  - *Are your professional and personal views of abortion and abortion restriction generally aligned?*

Thank you for sharing more about yourself.

**Section 2: Preparation**

I’d like to talk about the overturn of *Roe v. Wade* now. On May 2^nd^, 2022 a Supreme Court draft opinion indicating that *Roe v. Wade* would be overturned was leaked. I’d like to hear about the timeframe between May 2^nd^, 2022 when the draft opinion was leaked up to the official overturn on June 24^th^, 2022.

- *When did you become aware that Roe v. Wade might be overturned (if you were aware before the actual overturn)?*
  - *When did you learn that it had officially been overturned?*
- *After the Supreme Court opinion leak in May, how did your clinic prepare for a possible overturn, if at all?*
  - *What was your involvement in the clinic preparations, if any?*
- *After the Supreme Court opinion leak in May, how did your institution prepare for the possible overturn, if at all?*
  - *What was your involvement in institutional preparations, if any?*
- *After the Supreme Court opinion leak in May, how did you prepare for a possible overturn, if at all?*

This was helpful in understanding how you and your workplace learned about the overturn and your thoughts about the overturn itself.

**Section 3: Counseling/Practice Changes**

Now that we’ve discussed the overturn, I’d like to talk about your counseling prior to the overturn and how it has changed since, if at all.

- *Prior to the overturn of Roe v. Wade, what topics surrounding abortion did you typically discuss in your counseling (i.e. options other than abortion, waiting periods, procedure locations, legislation)?*
  - *How often did you discuss options other than abortion with patients (i.e. adoption)?*
  - *How often did you have to prepare for complex births?*
  - *How much time did you typically spend discussing the specific legislation in place, if any?*
- *What changes have you made to your counseling around abortion since the overturn of Roe v. Wade?*
  - *How has the timing of the abortion discussion changed, if at all?*
  - *What topics do you most frequently address in counseling?*
    - *How often do you discuss options other than abortion with patients now (i.e adoption)?*
    - *How often do you prepare for complex births now?*
    - *How much time do you typically spend discussing the specific legislation in place, if any?*
- **If they refer in-state:**
  - *Prior to the overturn, what did your conversations about abortion referrals within your institution and/or to in-state clinics involve?*
  - *Since the overturn, how have your conversations with patients within institution or in-state referrals changed?*
- **If they refer out-of-state**:
  - *Prior to the overturn, what did your conversations about out-of-state abortion referrals involve?*
  - *Since the overturn, how have your conversations with patients about out-of-state abortion referrals changed?*

I’d like to talk more specifically about workarounds now. We will define a workaround as a change to counseling or to the referral process to circumvent abortion restrictions and/or their effects. One example would be placing an abortion referral for a patient before the patient has confirmed a decision to terminate the pregnancy so that they can schedule early to avoid missing the legal window due to long waitlists. Another example would be writing up documentation differently to avoid the possibility of a patient becoming caught in litigation.

- *Prior to the overturn of Roe v. Wade, how often did you perform workarounds for abortion referrals, if at all?*
- *Since the overturn of Roe v. Wade, how often do you perform workarounds, if at all?*
- *What additional practices have you started or utilized more to increase accessibility of abortion, if any?(i.e. providing abortion fund resources)*
- Other changes to practice
  - *What changes have you made in documentation practices, if any?*

Thank you for helping me to understand what your counseling looked like both before and after the Roe overturn.

**Section 4: Ability to Fulfill Job Duties**

Next, I’d like to get an understanding of how well you feel that you are able to fulfill certain aspects of your role as a prenatal genetic counselor as well as your participation in advocacy efforts.

First, we’ll talk about non-directiveness, which I would like to define. We will define non-directiveness as the presentation of information without the encouragement of a particular action.

- *Prior to the overturn of Roe v. Wade, how would you describe your ability to be non-directive in your counseling?*
- *Since the overturn of Roe v. Wade, how has your ability to be non-directive in your counseling changed, if at all?*
  - If changed: *What specific factors affect your ability to be non-directive?*
- *Prior to the overturn of Roe v. Wade, in light of abortion legislation, how would you rate your confidence in communicating abortion options to patients on a scale of 1 to 10 with 1 representing not confident at all and 10 representing extremely confident?*
- *Since the overturn of Roe v. Wade, in light of abortion legislation, how would you rate your confidence in communicating abortion options to patients on a scale of 1 to 10 with 1 representing not confident at all and 10 representing extremely confident?*
  - If changed: *What specific factors have affected your confidence?*
- *Prior to the overturn of Roe v. Wade, did you feel that you were able to* ***keep up with changing abortion laws*** *in your state?*
  - *What challenges were there to keeping up with laws, if any?*
- *Since the overturn of Roe v. Wade, do you feel that you are able to* ***keep up with changing laws*** *in your state?*
  - *What sources do you utilize to find information?*
  - *Have you found information from professional organizations?*
  - *Does your institution distribute information to employees about changing policies?*
  - *How does this compare to before the overturn?*
- *Prior to the overturn of Roe v. Wade, what advocacy efforts had you engaged in regarding abortion restrictions in your state, if any?*
  - *What advocacy efforts have you engaged in within your institution if any?*
  - *What advocacy efforts have you engaged in with professional organizations?*
  - *What advocacy efforts have you engaged in with your state legislature?*
- *Since the overturn of Roe v. Wade, what advocacy efforts have you engaged in regarding abortion restrictions in your state, if any?*
  - *What advocacy efforts have you engaged in within your institution if any?*
  - *What advocacy efforts have you engaged in with professional organizations?*
  - *What advocacy efforts have you engaged in with your state legislature?*
  - **If increased advocacy:**
    - *Why did you increase your advocacy?*

Thanks for discussing these different areas of your role.

**Section 5: Job satisfaction/emotional wellbeing**

In this last section, we’ll focus on your job satisfaction before and after the overturn of *Roe v. Wade*.

- *Prior to the overturn of Roe v. Wade, how would you rate your job satisfaction on a scale of 1 to 10 with 1 representing not satisfied at all and 10 representing extremely satisfied?*
  - *Why did you choose that rating?*
- *Since the overturn of Roe v. Wade, how would you rate your job satisfaction on a scale of 1 to 10 with 1 representing not satisfied at all and 10 representing extremely satisfied?*
  - *Why did you choose that rating?*
  - *Since the overturn, have you considered switching specialties, professions, or retiring early due to changes in your role?*
- *Prior to the overturn of Roe v. Wade, on a scale of 1 to 10 how would you rate your satisfaction with your institution?*
  - *Why did you choose that rating?*
- *Since the overturn of Roe v. Wade, on a scale of 1 to 10 how would you rate your satisfaction with your institution?*
  - *Why did you choose that rating?*
- *Prior to the overturn of Roe v. Wade, how would you rate your satisfaction with your patient care with 1 representing not satisfied at all and 10 representing extremely satisfied?*
- *Since the overturn of Roe v. Wade, how would you rate your satisfaction with your patient care with 1 representing not satisfied at all and 10 representing extremely satisfied?*
- *Prior to the overturn of Roe v. Wade, how would you describe the atmosphere of your workplace and team?*
- *Since the overturn of Roe v. Wade, how would you describe the atmosphere of your workplace and team?*
- *Prior to the overturn of Roe v. Wade, how did you typically feel after coordinating an abortion for a patient?*
- *Since the overturn of Roe v. Wade, how do you typically feel after coordinating an abortion for a patient?*
  - *How does this compare to before the overturn?*
  - **If they refer out-of-state:** *How does this differ for in-state versus out-of-state referrals, if at all?*
- *Where do coworkers typically look for emotional support?*
  - *Where do you typically look for emotional support?*

We’ve reached the end of the interview, is there anything else that you would like to share that I didn’t ask about?

Thank you so much for your time today and going through all of these topics with me. This information will help us to better understand changes to prenatal genetic counseling practice across the United States in the wake of the overturn of Roe v. Wade.
