# Supplementary material for: Before and after: The impact of the Roe v. Wade overturn on prenatal genetic counseling practice
Source: J Genet Couns. 2025 Aug 5;34(4):e70088. doi: 10.1002/jgc4.70088 (PMC12323291; doi:10.1002/jgc4.70088)
Supplement: Supplementary file 3 [file JGC4-34-0-s002.docx]

Liz Hart UMGCP Research Survey Guide

Start of Block: Informed Consent

Q30 Research Participant Informed Consent

Study Title:  Before and After: examining prenatal genetic counselors' perceptions of the impact of the Roe v. Wade overturn on their practice (HUM00227742)

PI: Elizabeth Hart, BS (University of Michigan, Class of 2024)

You are invited to participate in a research study that is investigating the effects of the overturn of Roe v. Wade on prenatal genetic counseling practices. To assist with this, we will ask you to answer a series of questions regarding abortion referral and coordination practices pre- and post-overturn. Completion of this survey is estimated to take approximately 10-15 minutes.

This survey is broken up into 4 parts:

1. Demographics and practice setting

2. Abortion referral and coordination pre-Roe v. Wade overturn
3. Abortion referral and coordination post-Roe v. Wade overturn
4. Additional demographics

At completion of the survey, you will have the chance to enter a raffle, where 10 participants will
win a $15 Amazon gift card. To enter this raffle, we will collect your name and email address at
the completion of this survey. This information will not be linked to your survey responses. 

The last survey question will ask if you would be interested in participating in a semi-structured interview to further discuss changes to prenatal genetic counseling practice in regards to the overturn of Roe v. Wade. Between 12-18 participants will be selected to be interviewed, and these individuals will
receive $25 as compensation for their time. Completing this survey does not obligate you to an interview. If you indicate that you are interested in participating
in an interview, you will be asked to provide your name and email so we can contact you
with further information. Your contact information (name and email address) will only be recorded for the purpose of scheduling your interview, if applicable. We will protect the confidentiality of your research records by removing these personal identifiers from your survey response using a data de-identification process. Your personal identifiers will only be associated with your survey answers through a randomized ID number that is stored securely.

Participating in this study is completely voluntary. Even if you decide to participate now, you
may change your mind and stop at any time. If you have questions about this research study or
wish to obtain more information, you may contact Elizabeth (Liz) Hart at lizhart@umich.edu.

By clicking the button to continue with this survey, you give your consent to participate in this study.

End of Block: Informed Consent

Start of Block: Demographics 1

Q1 Participants will need to meet the following criteria in order to participate in this survey:

 -	Must be a certified or board-eligible genetic counselor practicing in the United States.
 -	Must be currently practicing as a prenatal genetic counselor who sees patients in clinic.

-	Must have been conducting prenatal genetic counseling sessions for at least one (1) year prior to participation.
 -Must provide referrals for and/or coordinate abortion care.

- I meet this criteria  (1) 

- I do not meet this criteria  (2) 

Skip To: End of Survey If Participants will need to meet the following criteria in order to participate in this survey:  -... = I do not meet this criteria

Q2 What state do you primarily practice in?

▼ Alabama (1) ... Wyoming (50)

Q3 How many years have you practiced as a prenatal genetic counselor? Round to the nearest year.

- 1-3 years  (1) 

- 3-5 years  (2) 

- 5-10 years  (3) 

- 10-15 years  (4) 

- 15-20 years  (5) 

- 20+ years  (6) 

Q4 How familiar are you with the laws regarding abortion in your state of practice?

- 1-Very familiar  (1) 

- 2-Familiar  (2) 

- 3-Somewhat familiar  (3) 

- 4-Not familiar at all  (4) 

End of Block: Demographics 1

Start of Block: Demographics 2

Q5 What type of institution do you practice in?

- Academic  (1) 

- Public non-religious  (2) 

- Public religious  (3) 

- Private non-religious  (4) 

- Private religious  (5) 

Display This Question:

If What type of institution do you practice in? = Public religious

Or What type of institution do you practice in? = Private religious

Q6 Religious affiliation of institution:

- Catholic  (1) 

- Lutheran  (2) 

- Seventh Day Adventist  (3) 

- Baptist  (4) 

- Methodist  (5) 

- Protestant  (6) 

End of Block: Demographics 2

Start of Block: Pre-overturn

Q7 Prior to the overturn of Roe v. Wade on June 24th, 2022 what type of pregnancy termination was offered at your institution, if any? Select all that apply.

- D&C  (1) 

- D&E  (2) 

- Induction of labor  (3) 

- None  (4) 

- I don't know  (5) 

Q8 Prior to the overturn of Roe v. Wade on June 24th, 2022 what was the typical number of abortion referrals you made per month?

- 0  (1) 

- 1-3  (2) 

- 3-5  (3) 

- 5-10  (4) 

- 10+  (5) 

Q9 Prior to the overturn of Roe v. Wade June 24th, 2022 what was the distance from your institution to the nearest abortion provider?

- 0-10 miles  (1) 

- 10-20 miles  (2) 

- 20-50 miles  (3) 

- 50-100 miles  (4) 

- 100-200 miles  (5) 

- 200+ miles  (6) 

- I don't know  (7) 

Q10 Prior to the overturn of Roe v. Wade on June 24th, 2022, how often did you make referrals within your own institution, to in-state clinics, and/or to out-of-state clinics?

|  | Never (1) | Rarely (2) | Sometimes (3) | Often (14) | Always (15) |
| --- | --- | --- | --- | --- | --- |
| I referred within my own institution (1) |  |  |  |  |  |
| I referred to an in-state clinic (2) |  |  |  |  |  |
| I referred to an out-of-state clinic (3) |  |  |  |  |  |

Display This Question:

If Prior to the overturn of Roe v. Wade on June 24th, 2022, how often did you make referrals within... = I referred to an out-of-state clinic [ Rarely ] 

Or Prior to the overturn of Roe v. Wade on June 24th, 2022, how often did you make referrals within... = I referred to an out-of-state clinic [ Sometimes ] 

Or Prior to the overturn of Roe v. Wade on June 24th, 2022, how often did you make referrals within... = I referred to an out-of-state clinic [ Often ] 

Or Prior to the overturn of Roe v. Wade on June 24th, 2022, how often did you make referrals within... = I referred to an out-of-state clinic [ Always ] 

Q11 Mark all reasons for referring out-of-state:

- Gestational age limits in your state of practice  (1) 

- The distance to out-of-state clinics was shorter than the distance to in-state clinics  (2) 

- Few or no clinics in-state  (3) 

- List any additional reasons:  (4) __________________________________________________

Display This Question:

If Prior to the overturn of Roe v. Wade on June 24th, 2022, how often did you make referrals within... = I referred to an out-of-state clinic [ Rarely ] 

Or Prior to the overturn of Roe v. Wade on June 24th, 2022, how often did you make referrals within... = I referred to an out-of-state clinic [ Sometimes ] 

Or Prior to the overturn of Roe v. Wade on June 24th, 2022, how often did you make referrals within... = I referred to an out-of-state clinic [ Often ] 

Or Prior to the overturn of Roe v. Wade on June 24th, 2022, how often did you make referrals within... = I referred to an out-of-state clinic [ Always ] 

Q12 What type of assistance did you provide finding out-of-state abortion clinics, if any? Select all that apply.

- I did not provide assistance finding a clinic  (1) 

- I provided the patient with a resource for finding an out-of-state abortion clinic  (2) 

- I determined the nearest out-of-state option for the patient  (3) 

- I contacted out-of-state providers and sent records  (4) 

Q13 Prior to the overturn of Roe v. Wade on June 24th, 2022 what was the typical wait time for an abortion appointment for your patients from date of referral to date of appointment?

- 0-7 days  (1) 

- 8-14 days  (2) 

- 15-21 days  (3) 

- 22-28 days  (4) 

- 28+ days  (5) 

Q14 Prior to the overturn of Roe v. Wade on June 24th, 2022, how often did you perform and/or help coordinate these abortion-related tasks?

|  | Never (1) | Rarely (2) | Sometimes (3) | Often (4) | Always (5) |
| --- | --- | --- | --- | --- | --- |
| State mandated counseling (1) |  |  |  |  |  |
| Required ultrasound (2) |  |  |  |  |  |
| Parental consent (3) |  |  |  |  |  |
| Disposal of fetal remains (required or optional) (4) |  |  |  |  |  |
| Identify supplemental funding sources (5) |  |  |  |  |  |
| Investigate insurance coverage (6) |  |  |  |  |  |
| Writing letters for insurance review (7) |  |  |  |  |  |

End of Block: Pre-overturn

Start of Block: Post-overturn

Q15 Between the overturn of Roe v. Wade on June 24th, 2022 and midterm elections on November 8th, 2022, what type of pregnancy termination was offered at your institute, if any? Select all that apply.

- D&C  (1) 

- D&E  (2) 

- Induction of labor  (3) 

- None  (4) 

- I don't know  (5) 

Q16 Between the overturn of Roe v. Wade on June 24th, 2022 and midterm elections on November 8th, 2022, what was the typical number of abortion referrals you made per month?

- 0  (1) 

- 1-2  (2) 

- 3-5  (3) 

- 6-10  (4) 

- 10+  (5) 

Q17 Between the overturn of Roe v. Wade on June 24th, 2022 and midterm elections on November 8th, 2022, what was the distance from your institution to the nearest abortion provider?

- 0-10 miles  (1) 

- 10-20 miles  (2) 

- 20-50 miles  (3) 

- 50-100 miles  (4) 

- 100-200 miles  (5) 

- 200+ miles  (6) 

- I don't know  (7) 

Q18 Between the overturn of Roe v. Wade on June 24th, 2022 and midterm elections on November 8th, 2022, how often did you make referrals within your own institution, to in-state clinics, and/or to out-of-state clinics?

|  | Never (1) | Rarely (2) | Sometimes (3) | Often (4) | Always (5) |
| --- | --- | --- | --- | --- | --- |
| I refer within my own institution (1) |  |  |  |  |  |
| I refer to an in-state clinic (2) |  |  |  |  |  |
| I refer to an out-of-state clinic (3) |  |  |  |  |  |

Display This Question:

If Between the overturn of Roe v. Wade on June 24th, 2022 and midterm elections on November 8th, 202... = I refer to an out-of-state clinic [ Rarely ] 

Or Between the overturn of Roe v. Wade on June 24th, 2022 and midterm elections on November 8th, 202... = I refer to an out-of-state clinic [ Sometimes ] 

Or Between the overturn of Roe v. Wade on June 24th, 2022 and midterm elections on November 8th, 202... = I refer to an out-of-state clinic [ Often ] 

Or Between the overturn of Roe v. Wade on June 24th, 2022 and midterm elections on November 8th, 202... = I refer to an out-of-state clinic [ Always ] 

Q19 Mark all reasons for referring out-of-state:

- Gestational age limits in your state of practice  (1) 

- The distance to out-of-state clinics was shorter than the distance to in-state clinics  (2) 

- Few or no clinics in-state  (3) 

- List any additional reasons:  (4) __________________________________________________

Display This Question:

If Between the overturn of Roe v. Wade on June 24th, 2022 and midterm elections on November 8th, 202... = I refer to an out-of-state clinic [ Rarely ] 

Or Between the overturn of Roe v. Wade on June 24th, 2022 and midterm elections on November 8th, 202... = I refer to an out-of-state clinic [ Sometimes ] 

Or Between the overturn of Roe v. Wade on June 24th, 2022 and midterm elections on November 8th, 202... = I refer to an out-of-state clinic [ Often ] 

Or Between the overturn of Roe v. Wade on June 24th, 2022 and midterm elections on November 8th, 202... = I refer to an out-of-state clinic [ Always ] 

Q20 What type of assistance did you provide finding out-of-state abortion clinics, if any? Select all that apply.

- I did not provide assistance finding a clinic  (1) 

- I provided the patient with a resource for finding an out-of-state abortion clinic  (2) 

- I determined the nearest out-of-state option for the patient  (3) 

- I contacted out-of-state providers and sent records  (4) 

Q21 Between the overturn of Roe v. Wade on June 24th, 2022 and midterm elections on November 8th, 2022, what was the typical wait time for an abortion appointment for your patients from date of referral to date of appointment?

- 0-7 days  (1) 

- 7-14 days  (2) 

- 14-21 days  (3) 

- 21-28 days  (4) 

- 28+ days  (5) 

- I don't know  (6) 

Q22 Between the overturn of Roe v. Wade on June 24th, 2022 and midterm elections on November 8th, 2022, how often did you perform and/or help coordinate these abortion-related tasks?

|  | Never (1) | Rarely (2) | Sometimes (3) | Often (4) | Always (5) |
| --- | --- | --- | --- | --- | --- |
| State mandated counseling (1) |  |  |  |  |  |
| Required ultrasound (2) |  |  |  |  |  |
| Parental consent (3) |  |  |  |  |  |
| Required disposal of fetal remains (4) |  |  |  |  |  |
| Identify supplemental funding sources (5) |  |  |  |  |  |
| Investigate insurance coverage (6) |  |  |  |  |  |
| Writing letters for insurance review (7) |  |  |  |  |  |

End of Block: Post-overturn

Start of Block: Demographics 3

Q23 How would you describe your professional stance on abortion?

- Abortion should be an option throughout the entire pregnancy  (1) 

- Abortion should be an option up to a certain point in pregnancy  (2) 

- Abortion should be an option in limited circumstances (i.e. rape, incest)  (3) 

- Abortion should not be an option during pregnancy  (4) 

- Prefer not to answer  (5) 

Q24 Which of the following best describes your gender identity?

- Agender  (1) 

- Genderqueer  (2) 

- Hijra  (3) 

- Man  (4) 

- Non-binary  (5) 

- Pangender  (6) 

- Questioning  (7) 

- Transgender  (8) 

- Two Spirit  (9) 

- Woman  (10) 

- Prefer to self-describe:  (11) __________________________________________________

- Prefer not to answer  (12) 

Q25 What is your present religious or spiritual affiliation, if any?

- Agnostic  (1) 

- Atheist  (2) 

- Bahá’í  (3) 

- Buddhist  (4) 

- Catholic  (5) 

- Evangelical Protestant  (6) 

- Hindu  (7) 

- Jain  (8) 

- Jewish  (9) 

- Latter-Day Saints/LDS  (10) 

- Mainline Protestant  (11) 

- Muslim  (12) 

- Non-religious  (13) 

- Orthodox Christian  (14) 

- Other Christian  (15) 

- Shinto  (16) 

- Sikh  (17) 

- Taoist  (18) 

- Other  (19) __________________________________________________

- Prefer not to answer  (20) 

End of Block: Demographics 3

Start of Block: Recontact

Q26 I am open to being contacted by the research team to complete a follow-up qualitative interview:

- Yes  (1) 

- No  (2) 

Q27 Would you like to be entered in a raffle for a $15 Amazon gift card for completing the survey? Your response will remain anonymous.

- Yes  (1) 

- No  (2) 

End of Block: Recontact
